# (*Z*)-Ethyl 2-benzyl­idene-3-oxobutano­ate

**DOI:** 10.1107/S1600536811024974

**Published:** 2011-06-30

**Authors:** Arif I. Ismiyev

**Affiliations:** a Baku State University, Z. Khalilov St. 23, Baku AZ-1148, Azerbaijan

## Abstract

The title mol­ecule, C_13_H_14_O_3_, adopts a *Z* conformation about the C=C double bond. In the crystal, weak inter­molecular C—H⋯O hydrogen bonds with phenyl –CH atoms functioning as donors and the carbonyl O atom of an ester group as acceptor are observed.

## Related literature

For applications of β-keto ester derivatives, see: Benetti *et al.* (1995 ▶); Simon *et al.* (2004 ▶).
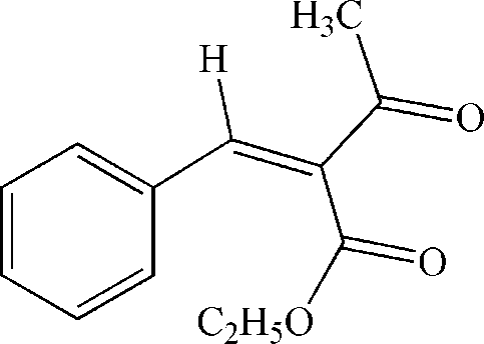


## Experimental

### 

#### Crystal data

C_13_H_14_O_3_
*M* *_r_* = 218.24
Orthorhombic, 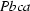

*a* = 7.8406 (5) Å
*b* = 16.8767 (12) Å
*c* = 17.5420 (13) Å
*V* = 2321.2 (3) Å^3^
*Z* = 8
Mo *K*α radiation
μ = 0.09 mm^−1^
*T* = 296 K
0.03 × 0.03 × 0.02 mm

#### Data collection

Bruker APEXII CCD diffractometer
Absorption correction: multi-scan (*SADABS*; Sheldrick, 1998) ▶ *T*_min_ = 0.997, *T*_max_ = 0.998
17709 measured reflections
2515 independent reflections
1459 reflections with *I* > 2σ(*I*)
*R*_int_ = 0.073

#### Refinement

*R*[*F* ^2^ > 2σ(*F* ^2^)] = 0.053
*wR*(*F* ^2^) = 0.125
*S* = 1.00
2515 reflections
147 parameters
H-atom parameters constrained
Δρ_max_ = 0.18 e Å^−3^
Δρ_min_ = −0.17 e Å^−3^

### 

Data collection: *APEX2* (Bruker, 2005 ▶); cell refinement: *SAINT-Plus* (Bruker, 2001 ▶); data reduction: *SAINT-Plus*; program(s) used to solve structure: *SHELXTL* (Sheldrick, 2008 ▶); program(s) used to refine structure: *SHELXTL*; molecular graphics: *SHELXTL*; software used to prepare material for publication: *SHELXTL*.

## Supplementary Material

Crystal structure: contains datablock(s) global, I. DOI: 10.1107/S1600536811024974/kp2335sup1.cif

Structure factors: contains datablock(s) I. DOI: 10.1107/S1600536811024974/kp2335Isup2.hkl

Supplementary material file. DOI: 10.1107/S1600536811024974/kp2335Isup3.cml

Additional supplementary materials:  crystallographic information; 3D view; checkCIF report

## Figures and Tables

**Table 1:** Hydrogen-bond geometry (Å, °)

*D*—H⋯*A*	*D*—H	H⋯*A*	*D*⋯*A*	*D*—H⋯*A*
C4—H4*A*⋯O2	0.93	2.62	3.544 (3)	170
C5—H5*A*⋯O2	0.93	2.65	3.412 (3)	140

**Fig. 1.**
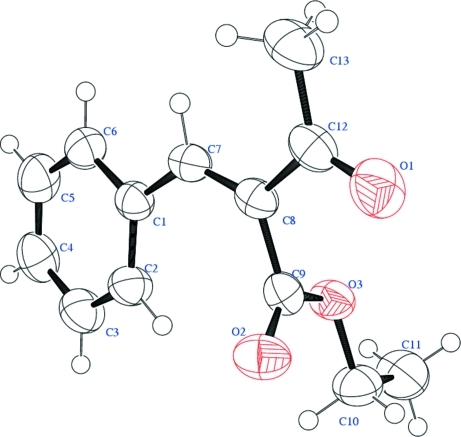
The molecular structure of the title compound, with the atomic numbering scheme. Displacement ellipsoids were drawn at the 30% probability level.

## supplementary crystallographic information

### Comment

β-Keto ester derivatives, as important synthetic intermediates, are widely applied in the synthesis of new heterocyclic derivatives presenting new pharmacological properties (Benetti *et al.*, 1995; Simon *et al.*, 2004). The molecular structure of the title compound adopts a *Z*-conformation at the carbon-carbon double bond (Fig. 1). The molecules are connected mainly by intermolecular C—H···O interactions (Table 1).

### Experimental

Benzaldehyde (20 mmol) and acetoacetic ester (20 mmol) were dissolved in 20 mL ethanol. After adding 0.50 mL piperidine mixture was stirred at room tempertue for 10 h. White crystals were obtained. The crystals were filtered off and washed with ethanol. Then they were dissolved in ethanol (20 mL) and recrystallised to yield colourless block-shaped crystals of the title compound.

### Refinement

All H atoms were positioned geometrically (C—H = 0.93–0.97 Å) and refined as riding, allowing for free rotation of the methyl groups. The constraint *U*_iso_(H) = 1.2 *U*_eq_(C) or 1.5 *U*_eq_(C) (methyl C) was applied.

### Crystal data

**Table:**

C_13_H_14_O_3_	*F*(000) = 928
*M**_r_* = 218.24	*D*_x_ = 1.249 Mg m^−^^3^
Orthorhombic, *P**b**c**a*	Mo *K*α radiation, λ = 0.71073 Å
Hall symbol: -P 2ac 2ab	Cell parameters from 1409 reflections
*a* = 7.8406 (5) Å	θ = 2.3–21.4°
*b* = 16.8767 (12) Å	µ = 0.09 mm^−^^1^
*c* = 17.5420 (13) Å	*T* = 296 K
*V* = 2321.2 (3) Å^3^	Prism, colourless
*Z* = 8	0.03 × 0.03 × 0.02 mm

### Data collection

**Table:**

Bruker APEXII CCD diffractometer	2515 independent reflections
Radiation source: fine-focus sealed tube	1459 reflections with *I* > 2σ(*I*)
graphite	*R*_int_ = 0.073
phi and ω scans	θ_max_ = 27.0°, θ_min_ = 2.3°
Absorption correction: multi-scan (*SADABS*; Sheldrick, 1998)	*h* = −10→10
*T*_min_ = 0.997, *T*_max_ = 0.998	*k* = −21→21
17709 measured reflections	*l* = −22→22

### Refinement

**Table:**

Refinement on *F*^2^	Primary atom site location: structure-invariant direct methods
Least-squares matrix: full	Secondary atom site location: difference Fourier map
*R*[*F*^2^ > 2σ(*F*^2^)] = 0.053	Hydrogen site location: difference Fourier map
*wR*(*F*^2^) = 0.125	H-atom parameters constrained
*S* = 1.00	*w* = 1/[σ^2^(*F*_o_^2^) + (0.0409*P*)^2^ + 0.8814*P*] where *P* = (*F*_o_^2^ + 2*F*_c_^2^)/3
2515 reflections	(Δ/σ)_max_ < 0.001
147 parameters	Δρ_max_ = 0.18 e Å^−^^3^
0 restraints	Δρ_min_ = −0.17 e Å^−^^3^

### Special details

**Table:**

Geometry. All e.s.d.'s (except the e.s.d. in the dihedral angle between two l.s. planes) are estimated using the full covariance matrix. The cell e.s.d.'s are taken into account individually in the estimation of e.s.d.'s in distances, angles and torsion angles; correlations between e.s.d.'s in cell parameters are only used when they are defined by crystal symmetry. An approximate (isotropic) treatment of cell e.s.d.'s is used for estimating e.s.d.'s involving l.s. planes.
Refinement. Refinement of *F*^2^ against ALL reflections. The weighted *R*-factor *wR* and goodness of fit *S* are based on *F*^2^, conventional *R*-factors *R* are based on *F*, with *F* set to zero for negative *F*^2^. The threshold expression of *F*^2^ > σ(*F*^2^) is used only for calculating *R*-factors(gt) *etc*. and is not relevant to the choice of reflections for refinement. *R*-factors based on *F*^2^ are statistically about twice as large as those based on *F*, and *R*- factors based on ALL data will be even larger.

### Fractional atomic coordinates and isotropic or equivalent isotropic displacement parameters (Å^2^)

**Table:**

	*x*	*y*	*z*	*U*_iso_*/*U*_eq_	
O1	0.3871 (2)	0.22540 (10)	0.50159 (10)	0.0679 (5)	
O2	0.4542 (2)	0.17527 (10)	0.66960 (9)	0.0577 (5)	
O3	0.66163 (18)	0.11601 (9)	0.60314 (8)	0.0488 (4)	
C1	0.3604 (3)	−0.03682 (12)	0.61668 (12)	0.0416 (5)	
C2	0.4421 (3)	−0.02544 (14)	0.68650 (12)	0.0511 (6)	
H2A	0.4778	0.0251	0.7004	0.061*	
C3	0.4706 (3)	−0.08813 (15)	0.73500 (14)	0.0599 (7)	
H3A	0.5266	−0.0798	0.7810	0.072*	
C4	0.4170 (3)	−0.16243 (15)	0.71591 (15)	0.0620 (7)	
H4A	0.4371	−0.2047	0.7488	0.074*	
C5	0.3338 (3)	−0.17479 (15)	0.64858 (16)	0.0634 (7)	
H5A	0.2960	−0.2254	0.6360	0.076*	
C6	0.3056 (3)	−0.11284 (14)	0.59944 (14)	0.0510 (6)	
H6A	0.2488	−0.1220	0.5538	0.061*	
C7	0.3282 (3)	0.02582 (13)	0.56098 (12)	0.0417 (5)	
H7A	0.2576	0.0111	0.5208	0.050*	
C8	0.3835 (3)	0.10083 (13)	0.55822 (11)	0.0403 (5)	
C9	0.5003 (3)	0.13555 (12)	0.61673 (12)	0.0410 (5)	
C10	0.7862 (3)	0.14424 (16)	0.65832 (14)	0.0585 (7)	
H10A	0.7593	0.1242	0.7087	0.070*	
H10B	0.7859	0.2017	0.6601	0.070*	
C11	0.9541 (3)	0.11498 (19)	0.63350 (16)	0.0738 (8)	
H11A	1.0395	0.1312	0.6695	0.111*	
H11B	0.9807	0.1365	0.5842	0.111*	
H11C	0.9518	0.0582	0.6306	0.111*	
C12	0.3325 (3)	0.15853 (15)	0.49856 (13)	0.0485 (6)	
C13	0.2141 (3)	0.13393 (17)	0.43558 (14)	0.0677 (8)	
H13A	0.1707	0.1802	0.4103	0.102*	
H13B	0.1209	0.1041	0.4565	0.102*	
H13C	0.2749	0.1017	0.3996	0.102*	

### Atomic displacement parameters (Å^2^)

**Table:**

	*U*^11^	*U*^22^	*U*^33^	*U*^12^	*U*^13^	*U*^23^
O1	0.0888 (13)	0.0502 (11)	0.0645 (11)	0.0025 (10)	−0.0006 (10)	0.0121 (9)
O2	0.0627 (11)	0.0574 (10)	0.0530 (10)	0.0107 (8)	0.0007 (8)	−0.0164 (8)
O3	0.0411 (8)	0.0639 (10)	0.0414 (9)	0.0030 (7)	−0.0003 (7)	−0.0061 (7)
C1	0.0414 (11)	0.0441 (13)	0.0392 (12)	0.0014 (10)	0.0031 (9)	−0.0016 (10)
C2	0.0671 (15)	0.0430 (13)	0.0433 (13)	−0.0046 (11)	−0.0060 (11)	0.0027 (11)
C3	0.0719 (17)	0.0594 (16)	0.0484 (15)	−0.0003 (13)	−0.0031 (13)	0.0102 (12)
C4	0.0742 (17)	0.0507 (15)	0.0609 (16)	0.0039 (13)	0.0113 (14)	0.0130 (13)
C5	0.0754 (18)	0.0447 (14)	0.0702 (18)	−0.0076 (13)	0.0139 (15)	−0.0028 (13)
C6	0.0534 (14)	0.0493 (14)	0.0501 (14)	−0.0056 (11)	0.0029 (11)	−0.0042 (12)
C7	0.0407 (11)	0.0483 (13)	0.0359 (11)	0.0035 (10)	−0.0023 (9)	−0.0031 (10)
C8	0.0379 (11)	0.0484 (13)	0.0347 (11)	0.0081 (10)	0.0010 (9)	−0.0003 (10)
C9	0.0455 (12)	0.0388 (11)	0.0386 (12)	0.0037 (10)	0.0046 (10)	0.0029 (10)
C10	0.0505 (14)	0.0740 (18)	0.0510 (14)	−0.0034 (13)	−0.0098 (11)	−0.0061 (13)
C11	0.0472 (15)	0.106 (2)	0.0685 (17)	0.0017 (15)	−0.0065 (13)	0.0004 (16)
C12	0.0501 (13)	0.0554 (15)	0.0401 (13)	0.0112 (12)	0.0055 (11)	0.0027 (11)
C13	0.0714 (18)	0.0825 (19)	0.0492 (14)	0.0105 (15)	−0.0126 (13)	0.0149 (14)

### Geometric parameters (Å, °)

**Table:**

O1—C12	1.208 (3)	C6—H6A	0.9300
O2—C9	1.200 (2)	C7—C8	1.339 (3)
O3—C9	1.329 (2)	C7—H7A	0.9300
O3—C10	1.455 (3)	C8—C12	1.484 (3)
C1—C6	1.386 (3)	C8—C9	1.495 (3)
C1—C2	1.395 (3)	C10—C11	1.472 (3)
C1—C7	1.462 (3)	C10—H10A	0.9700
C2—C3	1.376 (3)	C10—H10B	0.9700
C2—H2A	0.9300	C11—H11A	0.9600
C3—C4	1.364 (4)	C11—H11B	0.9600
C3—H3A	0.9300	C11—H11C	0.9600
C4—C5	1.365 (4)	C12—C13	1.502 (3)
C4—H4A	0.9300	C13—H13A	0.9600
C5—C6	1.373 (3)	C13—H13B	0.9600
C5—H5A	0.9300	C13—H13C	0.9600
			
C9—O3—C10	116.00 (17)	O2—C9—O3	124.3 (2)
C6—C1—C2	117.5 (2)	O2—C9—C8	124.4 (2)
C6—C1—C7	118.0 (2)	O3—C9—C8	111.27 (18)
C2—C1—C7	124.5 (2)	O3—C10—C11	107.1 (2)
C3—C2—C1	120.8 (2)	O3—C10—H10A	110.3
C3—C2—H2A	119.6	C11—C10—H10A	110.3
C1—C2—H2A	119.6	O3—C10—H10B	110.3
C4—C3—C2	120.3 (2)	C11—C10—H10B	110.3
C4—C3—H3A	119.8	H10A—C10—H10B	108.6
C2—C3—H3A	119.8	C10—C11—H11A	109.5
C3—C4—C5	120.0 (2)	C10—C11—H11B	109.5
C3—C4—H4A	120.0	H11A—C11—H11B	109.5
C5—C4—H4A	120.0	C10—C11—H11C	109.5
C4—C5—C6	120.2 (2)	H11A—C11—H11C	109.5
C4—C5—H5A	119.9	H11B—C11—H11C	109.5
C6—C5—H5A	119.9	O1—C12—C8	119.1 (2)
C5—C6—C1	121.2 (2)	O1—C12—C13	120.7 (2)
C5—C6—H6A	119.4	C8—C12—C13	120.3 (2)
C1—C6—H6A	119.4	C12—C13—H13A	109.5
C8—C7—C1	130.7 (2)	C12—C13—H13B	109.5
C8—C7—H7A	114.6	H13A—C13—H13B	109.5
C1—C7—H7A	114.6	C12—C13—H13C	109.5
C7—C8—C12	124.0 (2)	H13A—C13—H13C	109.5
C7—C8—C9	122.95 (19)	H13B—C13—H13C	109.5
C12—C8—C9	113.07 (19)		
			
C6—C1—C2—C3	1.7 (3)	C10—O3—C9—O2	1.5 (3)
C7—C1—C2—C3	−178.6 (2)	C10—O3—C9—C8	−177.80 (18)
C1—C2—C3—C4	−0.9 (4)	C7—C8—C9—O2	−97.9 (3)
C2—C3—C4—C5	−0.4 (4)	C12—C8—C9—O2	80.6 (3)
C3—C4—C5—C6	0.8 (4)	C7—C8—C9—O3	81.4 (2)
C4—C5—C6—C1	0.0 (4)	C12—C8—C9—O3	−100.1 (2)
C2—C1—C6—C5	−1.2 (3)	C9—O3—C10—C11	178.9 (2)
C7—C1—C6—C5	179.0 (2)	C7—C8—C12—O1	178.8 (2)
C6—C1—C7—C8	−171.0 (2)	C9—C8—C12—O1	0.3 (3)
C2—C1—C7—C8	9.3 (4)	C7—C8—C12—C13	−0.9 (3)
C1—C7—C8—C12	−177.4 (2)	C9—C8—C12—C13	−179.4 (2)
C1—C7—C8—C9	1.0 (4)		

### Hydrogen-bond geometry (Å, °)

**Table:**

*D*—H···*A*	*D*—H	H···*A*	*D*···*A*	*D*—H···*A*
C4—H4A···O2^i^	0.93	2.62	3.544 (3)	170
C5—H5A···O2^ii^	0.93	2.65	3.412 (3)	140

Symmetry codes: (i) ; (ii) .
